# Optimal Treatment Timing in Orthodontics: A Scoping Review

**DOI:** 10.1055/s-0043-1768974

**Published:** 2023-06-13

**Authors:** Mohammad A. Hamidaddin

**Affiliations:** 1 Department of Preventive Dental Sciences, College of Dentistry, King Faisal University, Al Ahsa, Saudi Arabia

**Keywords:** early orthodontic treatment, Early intervention, late treatment

## Abstract

The appropriate timing for orthodontic intervention has been a subject of debate for a long time, concerning not only the immediate effects but also the long-term benefits of such treatment. This review aimed to find the appropriate treatment timing for the intervention of various orthodontic problems. A literature search was performed in all major databases, including PubMed and Cochrane Library, until February 20, 2023. All observational and experimental studies published in English that compared early versus late orthodontic treatment in different types of orthodontic problems were included. Data selection and charting were undertaken by a single investigator. A total of 32 studies were identified that discussed various aspects of interventions, including Class II and Class III malocclusion, pseudo-Class III malocclusion, anterior and posterior crossbite, extractions, and long-term benefits. Overall, early intervention was not found to be superior in terms of effectiveness, overall duration of appliances, and cost–benefit ratio. Early intervention should be reserved for specific conditions or localized malocclusions that have psycho-social benefits, or to significantly reduce the severity of problems to be dealt with in comprehensive treatment in the permanent dentition.

## Introduction

The appropriate timing for initiation of orthodontic treatment has always been a subject of controversy and debate. Despite advancements in diagnostic tools and treatment approaches, there is still no consensus among orthodontists on the ideal timing for orthodontic intervention. 1 Early or phase I treatment refers to treatment done in primary or mixed dentition to intercept certain malocclusions so that the later treatment is easier, short in duration, and gives more stable results. On the other hand, single-phase treatment at the stage of early permanent dentition shortens the overall treatment duration, reduces cost, and causes less issues with “burnout,” more typically seen in two-phase treatment. 2

Early initiation of orthodontic treatment allows interception of the developing malocclusions and reduces their severity, which simplifies the second phase of orthodontic treatment. 3 The benefits of early treatment include improved dental health as crowding is relieved, allowing easy access to oral hygiene measures. Early correction of dentofacial irregularities has a positive psychological benefit, especially for those children who are bullied for their facial or dental appearance. Functional or orthopaedic appliances work better when given during the period of growth, and the late introduction of growth modulation has a negligible orthopaedic effect. Early identification and elimination of the contributing factors/etiology of malocclusion prevent aberrant growth and incorrect development of the jaw. 4

Although it is recommended by the American Association of Orthodontists that orthodontic screening starts before or at the age of 7 years, 5 many orthodontists prefer not to undertake treatment in the mixed dentition phase and postpone it until all the permanent teeth have erupted. They hold the belief that there is no significant difference in the ultimate treatment results whether the treatment is initiated early or late. Late treatment eliminates the need to compensate for unexpected variations due to residual growth. There is controversy regarding the long-term advantages of early treatment, and the available literature shows a focus mainly on the management of Class II malocclusions. 6 Less attention has been paid to other orthodontic problems like oral habits, anterior and posterior crossbite, tooth-size arch length discrepancies, eruption problems, extraction for orthodontic treatment, and maxillary arch expansion. Hence, this review aimed to find the appropriate treatment timing for intervention for various orthodontic problems.

## Methods

The present review was performed with identification of the research question, locating the relevant literature, selecting the eligible research, and collecting and analyzing the data. The search strategies in dental education and research described by Khurshid et al were followed. 7 An electronic search was conducted on the following databases and registers: PubMed/Medline, Embase, ISI Web of Science, Scopus, ClinicalTrials.gov, and CENTRAL. Gray literature, which included conference proceedings and unpublished literature, was searched via the library services and Google/Google Scholar. All publications until February 20, 2023 were included.

**Fig. 1:**
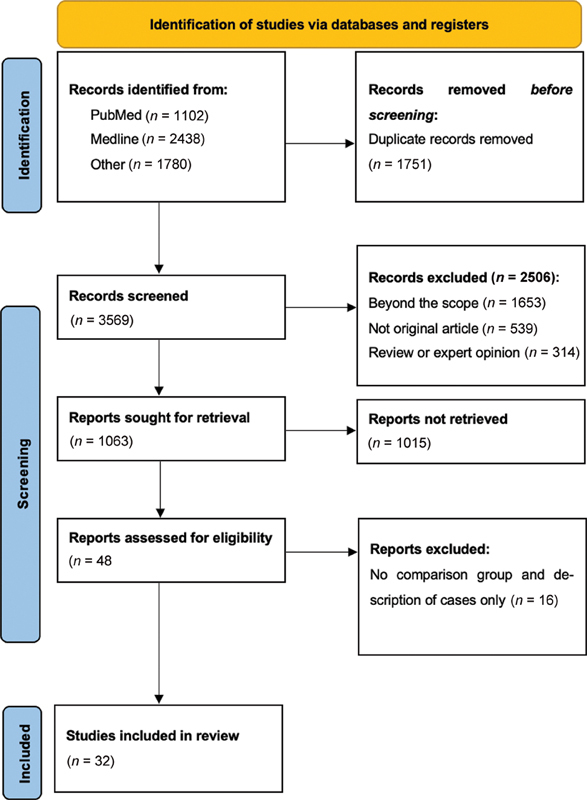
Flow diagram showing the process of selection of the studies in this review.

All clinical studies that evaluated early versus late treatment of any type of malocclusion were included and were screened for eligibility based on titles and abstracts. Only observational studies and clinical trials were included; case reports, reviews, opinions, and animal studies were excluded. To achieve a wide coverage, there were no restrictions on the publication date; however, the search was limited to English language only due to the lingual expertise of the author. The flowchart in Fig. 1 shows the selection process during the literature search. 8 Finally, 32 articles met the inclusion criteria, and the full texts were downloaded for the review process.

A form for charting the data was developed and information related to the type of article, publication year, study participants, and the outcome of the treatment, such as overall treatment time; cost–benefit ratio; skeletal, dental, and soft tissue changes; long-term retention; and incidence of trauma were extracted. The information extracted was analyzed using a qualitative content approach.

## Results

All published articles that compared early with late intervention of different kinds of malocclusions were reviewed. A total of 3,569 articles were retrieved from different databases after removing duplicates. We aimed for broader search terms to include all eligible articles ( Table 1 ). These publications were screened for the title and 1,063 articles were selected to review the title and abstract. The full text of 48 articles was downloaded and 16 were excluded for not meeting the eligibility criteria. Finally, this review found a total of 32 articles related to the topic under investigation, with a total sample size of 2,854 individuals and their age ranging from 7 to 15 years ( Table 2 ).

**Table 1:** Search strategy used in this review

Search Terms
#1 (“twin block” or bionator or activator or Frankel or “myofunctional appliance” or “functional appliance” or facemask or chincup or prognath* or retrognath* or deepbite or openbite or trauma or “serial extraction” or “premolar extraction” or class III or class II or malocclusion or orthodontic or “arch expansion” or crossbite or “maxillary expansion” or “palatal expansion”). #2 (“one phase” or “two phase” or early or timing or late). #1 AND #2 3062 results #1 AND #2 (English Language filter): 2538 results

**Table 2:** Summary of the findings of the included studies

Author/year	Type of orthodontic problem charted	Study design	Sample size	Age of participants	Treatment groups	Conclusion
O'Brien et al 2003 9 10	Class II	RCT	174	8–10 years (range)	Twin block in 8–10 years old children vs. untreated control	Dentoalveolar changes, (reduction of overjet and severity) with no significant skeletal change with early twin block appliance. Better self-esteem with early treatment
Dolce et al 2007 11	Class II	RCT	261	6.9–12.9 years (range)	Bionator vs. headgear/biteplane vs. untreated control	Early treatment with any appliance had no skeletal effect in long term (The long-term skeletal differences for all 3 groups were within 1 degrees)
Tulloch et al 1997 12	Class II	RCT	166	9.4 years (mean)	Headgear/bionator vs. untreated control	Short term significant skeletal changes with headgear or bionator (changes in ANB were 0.93 degrees, 1.07 degrees, and 0.17 degrees for bionator, headgear and control group)
Cha et al 2019 13	Class II	Observational	120	9.77–15.72 years (range)	Single phase vs. two phases treatment of Class II high angle cases	Early treatment has no skeletal or dental advantages over late treatment in high angle Class II
Bremen and Pancherz 2002 14	Class II	Observational	204	Chronological age not mentioned	Treatment of Class II Div 1 in early mixed, late mixed and permanent dentition	Late treatment of Class II was more efficient than intervention at early mixed or late mixed dentition. Permanent dentition group had a greater PAR score reduction (77%) than treated with functional appliances or a combination (60% for fixed appliances and 71% for functional or a combination)
Oh et al 2017 15	Class II	Observational	163	7–15 years (range)	Early vs. late treatment of Class II compared with untreated controls	Early treatment group has reduced need for extraction and reduced duration of full fixed appliance. (Extraction need of 5.6 vs. 37.9% in treatment and control group.) Late treatment group have less overall treatment time (33.7 vs. 53.1 months)
Baccetti et al 2000 16	Class II	Observational	79	9 years in early group and 11 years in late group (mean)	Early vs. late treatment of twin block with untreated controls	Late treatment (during or slightly after the pubertal peak) with twin block is optimum for class II The skeletal correction contributes to 67% of overall correction in late treatment group compared with 53% in the other
Faltin et al 17	Class II	Observational	23	9–11 years (range)	Early vs. late treatment of Class II with bionator	Late treatment at pubertal peak to be an appropriate timing for intervention with bionator (The long-term difference in the mandibular length was 5.1mm between the two groups)
Quintão et al 2006 18	Class II	Observational	38	9.5 years (treatment group) and 9.9 years (control group) (mean)	Early treatment with twin block vs. no treatment	Significant improvement in profile, retraction of upper lip, and anterior movement of soft tissue pogonion (ANB changes of 1.33 degrees in early group vs. 0.03 degrees in the control)
Singh et al 2010 19	Class II	Observational	48	11.17 years (mean)	Twin block treatment at CVM 1–2, 3–4, and 4–5 was compared	Optimum timing for twin block is during or slightly after the pubertal peak
Faltin et al 2003 17	Class II	Observational	58	9.1 years (mean)	Early or late bionator/activator followed by fixed orthodontics compared with untreated control	Late treatment at puberty is more effective (3.6mm of difference in mandibular length compared with the controls)
Pavoni et al 2017 40	Class II	Observational	46	9.9 years (mean age)	Early vs. late treatment with myofunctional appliance	Late treatment at puberty is more effective (ANB changes of 3.4 degrees in late treatment group vs. 1.6 degrees in early group)
Cha 2003 20	Class III	Observational	85	Mean age was 9.82, 11.31, and 13.07 years in the three groups	Patients at prepubertal, pubertal and postpubertal were treated with rapid maxillary expansion and facemask	Treatment at prepubertal or pubertal has more skeletal effect (skeletal changes contribute to 80.1% in prepubertal group, 84.0% in pubertal group, and 63.6% in postpubertal group)
Yüksel et al 2001 21	Class III	Observational	34	8 years, 2 months to 14 years, 3 months (range)	Early and late treatment with facemask	No significant difference between early and late treatment
Baccetti et al 2000 22	Class III	Observational	29	5 years, 5 moths to 10 years (range)	Early mixed dentition vs. late mixed dentition treatment with facemask	More favorable treatment changes in early mixed dentition (maxillary sagittal growth was nearly 4 times greater in the early group compared with the late)
Wendl et al 2017 23	Class III	Observational	38	Up to 9 years in early group. More than 9 but before the pubertal growth spurt in the late group	Early (<9 years) vs. late (>9 years) treatment with chincup	Early treatment produced more skeletal effect with less dentoalveolar changes (early treatment was successful in 74 and 67% of cases treated early and late, respectively)
Gu and Rabie 2000 24	Pseudo-Class III	Observational	36		Early treatment with no treatment	Sufficient space for the eruption of canines and premolars in early treatment group (treatment resulted in 4.7 mm space gain in the upper arch)
Haruki et al 1998 25	Extraction	Observational	83	Mean age of 11 years, 3 months in early group and 13 years, 4 months in late group	Long-term stability was compared between early and late treatment	Early treatment is more stable, shows better results, increases the post treatment stability of mandibular incisor
O'Shaughnessy et al 2011 26	Extraction	Observational	100	Mean age of 7.95 years in serial extraction group	Serial extraction is compared with late premolar extraction and fixed treatment	Similar outcomes but active treatment time is less in early extraction
Wilson et al 1999 27	Extraction	Observational	88	Mean age of 9 years, 9 months in serial extraction no treatment group and 9 years 6 months in serial extraction with treatment group	Serial extraction with or without fixed orthodontic treatment and late premolar extraction	No significant difference in soft tissue profile changes
Wagner and Berg 2000 28	Extraction	Observational	40	Mean age of 8.2 years in serial extraction and 11.6 years in late extraction	Serial extraction vs. premolar extraction in the permanent dentition	Treatment duration was less with serial extraction; however, there was a long observation period (3.6 vs. 6 years)
Brin and Bollen 2011 29	Extraction	Observational	48	Mean age of 12.4 years in serial extraction and 12.8 years in late extraction	Serial extraction vs. premolar extraction in the permanent dentition	No difference in the incidence of external apical root resorption
Baccetti et al 2001 30	Posterior crossbite	Observational	62	Mean age of 11 years in early group and 13 years in late group	Hass appliance before and after the peak growth	Favors early treatment in terms of stability of results
Petrén and Bondemark 2008 31	Posterior crossbite	RCT	60	8.3–9.1 years	rapid maxillary expansion with quad helix is compared with expansion plate, composite only and no treatment	rapid maxillary expansion with quad helix (in mixed dentition) is superior to expansion plate and no treatment (quad helix took 4.8 months whereas expansion plate required 9.6 months in average to correct crossbite)
Sari et al 2003 32	Posterior crossbite	Observational	51	Mean age of 9.2 years in early group and 12.7 years in the late group	Rapid maxillary expansion in the mixed vs. permanent dentition	No significant advantage of early treatment
Mohan et al 2016 33	Posterior crossbite	Observational	54	Mixed to permanent dentition	Long-term stability of rapid palatal expansion in the mixed dentition vs. the permanent dentition	No difference in the intermolar distance in pts treated at mixed or permanent dentition
Bicakci et al 2005 34	Posterior crossbite	Observational	44	Mean age of 11 years, 8 months in early group and 12 years, 6 months in late group	Rapid maxillary expansion before and after pubertal peak	No significant difference between two groups in terms of nasal airway changes
Lippold et al 2013 35	Posterior crossbite	RCT	66	7.3 years (mean)	Functional unilateral crossbite in late deciduous or early mixed dentition vs. no treatment	Significant improvement in dental occlusion in early treatment in transverse dimensions of the maxilla, palatal depth, maxillary arch length and inclination, the midline deviation, the overjet and the overbite
Baccetti et al 2012 36	Deep bite	RCT	58	Mean age of 10.7 years in early group and 12.3 years in late group	Early (prepubertal) vs. late (pubertal) group	Treatment at puberty (permanent dentition) leads to significantly more favorable outcomes than treatment before puberty (3.1 vs. 1.4mm reduction)
Franchi et al 2011 37	Deep bite	Observational	58	9.7 years (mean)	Early treatment compared with untreated control	No advantage from early treatment
Chen et al 2011 38	Trauma	RCT	261	9.6 years in bionator, 9.7 in headgear, and 9.5 years in the late treatment	Early (headgear/biteplane or bionator) vs. late treatment	Minor benefits from early treatment. No significant difference in the incidence of the injury. Need to evaluate cost–benefit ratio
Koroluk et al 2003 39	Trauma	RCT	179	7.9–12.6 years (range)	Early vs. late (mixed dentition vs. permanent dentition)	No difference in the incidence of injury, need to see cost–benefit ratio

Abbreviation: RCT, randomized controlled trial.

### Class II Malocclusion

Early treatment of skeletal Class II has been extensively studied in the literature. Tulloch et al found significant short-term skeletal changes in growing Class II patients in mixed dentition with headgear and a bionator. Greater changes in the maxilla and mandible were found with headgear and a bionator, respectively, when compared with untreated controls. 12 On the other hand, clinical trials conducted by O'Brien et al 9 and Dolce et al 11 comparing one-phase treatment with two-phase treatment of skeletal Class II malocclusions concluded there was no statistical difference between the two groups in terms of final overjet, ANB angle reduction, and peer assessment rating (PAR) score. The two-phase treatment, with a greater number of follow-up visits, was not found to have a better outcome, and they favored late single-phase treatment. Baccetti et al 16 and Singh et al 19 concluded that the ideal timing for a Class II correction with the twin-block appliance is at or following the stage where the mandible reaches peak pubertal growth. Similarly, Faltin et al, 17 Pavoni et al, 40 and Franchi et al 37 found the pubertal peak to be an appropriate timing for intervention with an activator/bionator.

Cha et al compared the skeletal and dental outcomes of early versus late treatment for high-angle Class II cases and found no additional advantages in early treatment. 13 Similarly, management of Class II malocclusions was found to be more efficient when performed as a phase II treatment with fixed functional appliances, compared with removable appliances followed by fixed. 14 On the contrary, with phase I treatment, Oh et al found a reduced need for extractions, 15 and O'Brien et al 10 found psychosocial benefits, with improved self-esteem of the patients.

### Class III

In an observational study by Cha et al, early treatment with a facemask at the prepubertal or pubertal period resulted in more skeletal and less dentoalveolar advancement, whereas in the post-pubertal period, the majority of the advancement achieved was contributed by the dentoalveolar effect. 20 Similarly, Yüksel et al compared the treatment effect of a facemask initiated at the early and late growth stages. With cephalometric superimposition, they concluded that in both groups, forward movement of the maxilla was noted, with no statistically significant difference. 21 Moreover, reverse twin-block and a facemask were also investigated in an observational study comparing their effect in early and late mixed dentition. It concluded the facemask to be superior to reverse pull twin-block and the late mixed dentition stage to be appropriate for facemask therapy. 41

In contrast, another study by Baccetti et al found early mixed dentition to be suitable for overall craniofacial outcome when compared with late mixed dentition, although treatment at both stages produced significant maxillary growth and restrainment of the growth of the mandible. 22 With chincup therapy, Wendl et al noted treatment before the age of 9 years could produce more skeletal effects when compared with later treatment. 23

### Pseudo-Class III

In a controlled study by Gu and Rabie, treating pseudo-Class III early, with simple fixed appliances, created sufficient space for canine and premolar eruption, in addition to the correction of an anterior crossbite, when compared with untreated control. 24 They also noted an increase in arch width when the maxilla was relieved of entrapment early.

### Extractions

In an observational study, Haruki and Little found that compared with late treatment, early orthodontic treatment with first premolar extraction is more stable, shows better results, and increases the post-treatment stability of mandibular incisors. 25 On the other hand, both groups were found to have similar occlusal outcomes when assessed with PAR. 26 Similarly, when long-term changes of the soft tissue profile were compared in patients with early serial extraction and phase II premolar extraction, no significant changes were noted. 27 The advantage of serial extraction is a shorter period requiring a fixed appliance, but the total treatment duration, including the observation period, is longer. 28 Similarly, the amount of external apical root resorption was found to be comparable in either approach. 29

### Posterior Crossbite

Baccetti et al studied the effect of a Hass appliance at different skeletal maturity levels and concluded that skeletal expansion initiated before peak pubertal growth produces more skeletal expansion than that after peak pubertal growth. 30 Similarly, early treatment of a unilateral functional crossbite in late deciduous or early mixed dentition was found to result in favorable dental occlusion. 35 A randomized controlled trial found quad-helix superior for correcting a posterior crossbite over a removable expansion plate and composite only and no treatment. 31 No spontaneous correction of posterior crossbite was observed in the untreated controls. On the other hand, an investigation to compare the effect of an acrylic bonded rapid expander on mixed and permanent dentition did not show any significant difference in the skeletal and dental outcomes, suggesting no additional benefits of early treatment. 32

The long-term stability of the rapid expansion of the maxilla was compared by Mohan et al between two groups: one at mixed dentition and another at permanent dentition. 33 No significant differences in the stability of the intermolar width were found between the two groups, suggesting no added advantage of early expansion for retention and stability. Rapid maxillary expansion has the added advantage of increasing the nasal volume, decreasing nasal airflow resistance, and improving nasal respiration. Expanding the maxilla before the pubertal peak leads to a greater increase in the nasal minimum cross-sectional area than expansion initiated after the peak; however, the difference was not statistically significant. 34

### Deep Bite

A prospective clinical study by Franchi et al assessed the long-term outcome of the two-phase treatment of deep bite and compared it with untreated controls. 17 They found that early treatment of deep bite had no significant effect on the mandibular ramus growth or the posterior dentoalveolar segment in the vertical dimension; however, a significant increase in upper and lower incisor inclination was observed in the treated group.

In a clinical trial that compared the outcomes of deep bite management with bite plane and fixed appliances in patients at the prepubertal versus pubertal stage, Baccetti et al found no significant advantage of phase I therapy in the vertical dimension of ramus or posterior dentoalveolar segment. 36 They concluded that deep bite management is best performed at puberty.

### Trauma

Based on a clinical trial, Koroluk et al concluded that early treatment prevented trauma to the patients with an increased overjet in Class II Division 1 cases. 39 Most of the trauma events were minor and the advantages of early treatment did not outweigh the cost associated with it. Similar results were obtained in another trial by Chen et al, suggesting early treatment might be unfavorable based on the cost–benefit ratio. 38

## Discussion

### Class II Malocclusion

Class II malocclusions are a common type of malocclusion, with an overall global prevalence of 19.56%. The highest prevalence was reported for Caucasians (22.9%). 42 Children with Class II division 1 malocclusions often have a facial appearance that makes them vulnerable to teasing and bullying, directly affecting quality of life. 43 44 45 46 Prominent maxillary incisors are also vulnerable to various traumatic injuries.

Early treatment of Class II division 1 malocclusions has been popular in some parts of the world. Early intervention for Class II malocclusions involves the use of orthopaedic appliances like headgear to restrict the growth of the maxilla or functional appliances to stimulate mandibular growth. Early treatment with these approaches favors early improvement of the profile, along with correction of abnormal functions and perioral muscle activity. 47 A normal overjet and overbite can be achieved along with the alignment of the incisors, improving smile esthetics. Early treatment also helps to manage arch length discrepancies, directly improving crowding and securing space for erupting canines and premolars. The early phase treatment is planned to last for a short duration, most often with headgear or twin-block, with or without sectional fixed orthodontic appliances. The active phase is followed by a period of retention, where no active appliances are placed in the oral cavity; however, some passive appliances, like a transpalatal arch, Nance palatal button, lingual holding arch, or removable acrylic plates may be suggested to prevent relapse or mesial migration of molars, avoiding the loss of leeway space. Comprehensive orthodontic treatment can be initiated after all the permanent dentitions have erupted to treat any residual discrepancies.

Another approach in the management of Class II malocclusions is intervention in late mixed or early permanent dentition, where functional appliances are prescribed followed by fixed appliances with no period of retention in between. The transition to the full fixed appliance can be facilitated by the use of upper anterior inclined planes. 48 The advantages of the single-phase approach include decreased overall treatment duration and thus less chances of burn out, less cost, and no need for a retention phase within the active stages. 13

### Class III Malocclusion

Class III malocclusions clinically present with the mandibular dentition in a more forward position than the maxillary dentition, which can be due to a deficient maxilla or excessive mandible or both, which are often hereditary traits. The global prevalence of Class III malocclusions is approximately 6%, 42 with almost three times higher occurrence in Asians than in blacks or Caucasians. 49 Class III cases pose a unique challenge to clinicians, and a true skeletal base Class III must be distinguished from dental anterior crossbite and pseudo-Class III malocclusions.

Early treatment of Class III malocclusions has been utilized by many clinicians with varying degrees of success. The most important advantage of early Class III treatment is the reduction in the severity of the discrepancy, which lessens the complexity of the malocclusion and reduces the need for surgical intervention. No strong evidence exists in favor of intervention in deciduous dentition for Class III malocclusion. 50 Early treatment of Class III in mixed dentition can be done with appliances like the Frankel functional regulator (FR-3) or orthopaedic appliances like a facemask or chincup, depending on the nature of the skeletal imbalance.

Class III malocclusions presenting with maxillary deficiency have often been treated with protraction facemasks, which aim for forward displacement of the maxilla, enhancing growth at the sutures. Early treatment is recommended to encourage maxillary skeletal and dentoalveolar growth to synchronize with the growth of the mandible before active adolescent growth ceases. Initiating treatment later will mean no ability to utilize growth-related adaptations. McNamara et al recommend intervention early, during the exchange of the upper central incisors, which coincides with the cervical vertebrae stage CS1. 51 This corresponds to a chronological age of 7 to 8 years.

Kim et al concluded from a meta-analysis that facemasks produce skeletal changes only if the treatment is initiated before the age of 10 years, along with the use of expansion appliances in the initial period. 52 Similarly, a systematic review by Miao et al included 19 trials and concluded that facemask therapy is more successful in early mixed dentition than late mixed dentition. 53 However, another meta-analysis by Wang et al 54 did not find any difference in the maxillary growth, intermaxillary relationship, or incisor inclination when a protraction facemask was given to patients in the early mixed or late mixed dentition period.

Skeletal Class III malocclusions as a result of mandibular prognathism are extremely difficult to manage during the growth stage due to the difficulty in predicting future mandibular growth. A chincup prescribed during the growth stage rotates the mandible downward and backward, resulting in a relative decrease in the sagittal discrepancy, in addition to the lingual tipping of the lower incisors. 55 Chincup therapy is effective when initiated during the primary or early mixed dentition stage 56 however, the long-term stability of the result is unclear due to the risk of relapse owing to the return of the original growth pattern after active appliance therapy. 57 Early intervention with a chincup and correction of the anterior crossbite are believed to have preventive effect on the unfavorable sagittal discrepancy; however, there is no strong evidence to support this. 56 57

### Pseudo-Class III Malocclusion

This is a Class III malocclusion due to the forward shift of the mandible to achieve a maximum intercuspation owing to occlusal interferences that prevent posterior occlusion. 5 The most often noticed interferences are retroclined maxillary incisors and proclined mandibular incisors. A careful evaluation of the occlusion with the mandible in centric relation is required to diagnose pseudo-Class III malocclusions. Early intervention of this condition eliminates the anterior mandibular displacement, unlocking the maxillary incisors and allowing unrestricted maxillary growth. Early intervention guides the eruption of the canines and premolars into a Class I relationship, with a corrected mandibular position. 59 On the other hand, delaying treatment of pseudo-Class III may lead to collapse of the maxillary arch, loss of self-esteem, and structural damage of the associated tooth and the periodontium, leading to a true skeletal Class III malocclusion later. 60 61 An observational study involving 25 patients with 5-year follow-up after early correction of pseudo-Class III malocclusions with 2 × 4 appliances revealed that the treatment results were maintained in all the patients. 62

### Crowding Correction with Extraction

Severe crowding with tooth size arch length discrepancy is managed with extractions. Removal of some teeth can be done at an early age with serial extraction so that the severity of the malocclusion is reduced, or the extraction is delayed until all permanent dentitions (except third molars) have erupted. Most of the articles included in this review concluded that the final outcome showed no significant difference except in the length of overall treatment time. Early extraction needs to be closely observed to guide the eruption of the canines and premolars, and thus the observation period is longer. Early treatment is also believed to have a lower incidence of relapse. 63

### Posterior Crossbite

Posterior crossbite is the abnormal relationship of the posterior teeth in the transverse dimension. The prevalence of posterior crossbite ranges from 4 to 17% around the globe. 64 It can be unilateral or bilateral; skeletal, dental, or a combination of both. A unilateral posterior crossbite is often the result of bilateral maxillary constriction that leads to mandibular shift to one side to get maximum intercuspation. 65 Asymmetry in the face, with the chin deviated to the side of the crossbite, is often observed in such cases.

Posterior crossbite often does not correct by itself, but rather worsens during the later stage of occlusal development, with asymmetric strain on the muscles of mastication. 66 Hence, it needs to be corrected as soon as possible to restore the normal growth and development of the orofacial structure. The common causes of posterior crossbite include a digit sucking habit, mouth breathing, and abnormal swallowing patterns, which need to be ruled out before planning any intervention to correct the crossbite. Early treatment of posterior crossbite improves dental, skeletal, and functional relationships. Early treatment can be performed with fixed or removable expansion appliances that widen the maxillary arch by opening the mid-palatal suture. 67

### Deep Bite

Although deep bite is a common malocclusion seen in routine clinical practice, it is difficult to treat and retain. An overbite of 2 to 4 mm or 5 to 25% of the overlap of the lower incisors can be considered normal, and 25 to 40% overlap without any associated temporomandibular joint problems can be acceptable. 68 Overlap beyond 40% is considered deep bite, and has potential deleterious effects on the periodontium.

The etiology of deep bite is complex and contributed to by genetic and environmental factors. For convenience in management, deep bite can be divided into skeletal and dental deep bite. Early treatment of deep bite is believed to achieve dentoalveolar growth and increase posterior facial height, bringing about positive muscular adaptation. Treatment during growth results in a better skeletal relationship. On the other hand, failure to address deep bite leads to poor periodontal health. 69 Impingement of the mandibular incisor on the palatal tissue leads to palatal bone loss and labial migration of the maxillary incisors. Deep bite can sometimes lead to interference with the normal closing pattern of the mandible. 70

### Prevention of Trauma

Children and adolescents are very prone to traumatic dental injuries, which have long-term esthetic and psychological consequences, and thus prevention is highly beneficial. The global prevalence of such trauma has been estimated to exceed 15% in permanent dentition and up to 18% of children below 12 years old are affected. 71 Although different factors like gender, anterior open bite, neuromuscular disorders, and obesity contribute to the trauma, increased overjet is significantly associated and contributes to 21% of cases worldwide. 72

### Limitations of the Study

This was a scoping review with the broad aim of identifying the appropriate treatment timing for different types of malocclusions with different types of appliances. Being broad, this review was unable to explain the effect of individual type of appliances at different points in time in different conditions. A systematic review that focuses on individual malocclusions may provide in-depth information in this area of the orthodontic literature.

## Conclusions

The optimal time for the intervention of malocclusions remains debatable. There are multiple factors that affect the choice in the timing of the treatment, such as the amount and severity of skeletal discrepancy, growth potential, patient cooperation, financial affordability, psychological considerations, and estimated total treatment time. Early treatment should be reserved to situations where it may effectively cure some malocclusions, helping to either lessen or perhaps eliminate the need for complicated and costly treatments throughout puberty, instances where it has the potential to improve psychosocial status, and certain malocclusion where there is a higher risk for dental trauma.
